# Impact of Epithelial–Mesenchymal Transition on the Immune Landscape in Breast Cancer

**DOI:** 10.3390/cancers13205099

**Published:** 2021-10-12

**Authors:** Fatima-Zohra Khadri, Marianne Samir Makboul Issac, Louis Arthur Gaboury

**Affiliations:** 1 Institute for Research in Immunology and Cancer (IRIC), Université de Montréal, Montréal, QC H3T 1J4, Canada; fatima.zohra.khadri@umontreal.ca (F.-Z.K.); marianne.issac@umontreal.ca (M.S.M.I.); 2 Department of Pathology and Cell Biology, Faculty of Medicine, Université de Montréal, Montréal, QC H3T 1J4, Canada; 3 Department of Clinical and Chemical Pathology, Faculty of Medicine, Cairo University, Cairo 11956, Egypt

**Keywords:** epithelial–mesenchymal transition (EMT), tumor microenvironment (TME), immune landscape, inflammatory infiltrate, breast cancer, biomarkers, protein expression, immunohistochemistry

## Abstract

**Simple Summary:**

The process of epithelial–mesenchymal transition (EMT) is thought to influence breast cancer tumor progression by affecting both tumor cells and the tumor microenvironment (TME). We aimed to study the impact of EMT-related markers on a breast cancer cohort and specifically analyze the involvement of Snail, Twist, ZEB1, N-cadherin, Vimentin, GRHL2, E-cadherin, and EpCAM and their respective outcome on both immune infiltration of the TME and clinicopathological features. The inflammatory infiltrate was more often associated with poorly differentiated carcinomas including triple-negative breast cancer (TNBC). The altered pattern of protein expression of epithelial markers markedly influenced the magnitude of the inflammatory infiltrate found in the TME. Overall, our data highlight the potential beneficial association of the EMT signature with the immune inflammatory response. This may open new avenues for rational decision making in the clinical use of immunotherapy in subsets of breast cancer patients, specifically TNBC.

**Abstract:**

The impact of epithelial–mesenchymal transition (EMT) signature on the immune infiltrate present in the breast cancer tumor microenvironment (TME) is still poorly understood. Since there is mounting interest in the use of immunotherapy for the treatment of subsets of breast cancer patients, it is of major importance to understand the fundamental tumor characteristics which dictate the inter-tumor heterogeneity in immune landscapes. We aimed to assess the impact of EMT-related markers on the nature and magnitude of the inflammatory infiltrate present in breast cancer TME and their association with the clinicopathological parameters. Tissue microarrays were constructed from 144 formalin-fixed paraffin-embedded invasive breast cancer tumor samples. The protein expression patterns of Snail, Twist, ZEB1, N-cadherin, Vimentin, GRHL2, E-cadherin, and EpCAM were examined by immunohistochemistry (IHC). The inflammatory infiltrate in the TME was assessed semi-quantitatively on hematoxylin and eosin (H&E)-stained whole sections and was characterized using IHC. The inflammatory infiltrate was more intense in poorly differentiated carcinomas and triple-negative carcinomas in which the expression of E-cadherin and GRHL2 was reduced, while EpCAM was overexpressed. Most EMT-related markers correlated with plasma cell infiltration of the TME. Taken together, our findings reveal that the EMT signature might impact the immune response in the TME.

## 1. Introduction

Breast cancer is a highly heterogenous and complex disease characterized by a wide range of pathological and clinical features, unique morphological characteristics, distinct molecular subtypes, and varying responses to treatment [1]. Tumor progression is thought to be driven by the process of epithelial–mesenchymal transition (EMT), which enables epithelial cells to acquire mesenchymal features [2,3]. EMT is brought about by a switch in the expression patterns of crucial genes, thus initiating a cascade of molecular, cellular, and morphological changes in cells [4]. During EMT, epithelial cells lose their apical–basal polarity and intercellular junctions, along with the acquisition of a mobile and invasive phenotype, and a concomitant increase in cell self-renewal and emergence of heterogeneous subpopulations [5,6]. EMT is a multistep dynamic process allowing carcinoma cells to reside in various phenotypic states along the epithelial–mesenchymal (E–M) spectrum [7,8]. It is a reversible process, insofar as cells which undergo EMT can also undergo MET [8,9]; this dynamic reversibility has been coined epithelial-to-mesenchymal plasticity [10]. Previous work on breast cancers indicated that EMT is one of the most crucial biological processes inducing stem-cell properties [11]. Apart from chemoresistance [12] and immune evasion [13], EMT encompasses two of the most fundamental properties present in metastasis: invasiveness and stemness [8,9]. It has been reported that a hybrid E/M phenotype drives tumor initiation, allowing cell plasticity to differentiate into several lineages. The aggressive hybrid double-positive CD24^+^/CD44^+^ E/M cells display enhanced plasticity, metastatic capability, and stemness when compared to the fully epithelial CD24^+^/CD44^−^ cells or mesenchymal CD24^−^/CD44^+^ cells [8,9]. This “partial E/M” state has been linked to collective migration, providing new insights into the relationships among tumor budding, cancer cell migration, and altered EMT marker expression using a simple novel technique for the 3D assessment of the human tumor–host interface [6]. In their landmark study, Godin et al. proposed a morphological approach, using sequential chromogenic immunohistochemical multiplexing, which allows detection and quantification of cancer cells endowed with the hybrid E/M phenotypes, thus supporting the use of a hybrid E/M score as a promising prognostic biomarker for cancer patients [8].

In addition to its dramatic effect on tumor cells, EMT produces considerable changes in the dynamic landscape of the tumor microenvironment (TME). In the early stages of transformation, cytokines/chemokines secreted from tumor cells attract various immune and stromal cells into the TME [14,15]. Later, the ensuing influx of immune cells provides a conducive niche which fosters tumor progression, invasion, and metastasis. Thus, immune cells may play an important role in determining the clinical outcome of the disease and the response of the tumor to immuno- and chemotherapy [16]. A previous study using human breast cancer cell lines and animal models showed that EMT promotes the occurrence of an immune-suppressive TME [17].

EMT is an exceedingly complex phenomenon involving multiple components such as extrinsic factors, signaling pathways, transcription factors, and target genes. One key event in EMT is the so-called “cadherin switch” that leads to E-cadherin downregulation along with paralleled N-cadherin upregulation [18]. This is facilitated by several transcription factors such as Snail, Twist, and ZEB1 [19,20,21,22]. GRHL2 suppresses EMT via the direct repression of ZEB1 expression and inhibition of the TGF-β pathway [23], whereas EpCAM is involved in cadherin-dependent cell adhesions [24]. As for Vimentin, it is thought to modulate tumor cell migration and to contribute to angiogenesis [25].

The impact of the EMT signature on the recruitment of inflammatory cells into the TME of breast cancer is incompletely understood. Given the growing interest in the useof immunomodulatory drugs for the treatment of subsets of breast cancer patients, it is of major importance to understand the underlying tumor characteristics which dictate the inter-tumor heterogeneity in immune landscapes and to utilize this knowledge for rational decision making in the clinical use of immunomodulatory strategies [26].

Therefore, we sought to investigate the phenomenon of EMT as it relates to the inflammatory infiltrate present in breast cancer. In particular, we wanted to (1) characterize the phenomenon of EMT in breast cancer by analyzing the expression patterns of specific transcription factors linked to EMT (Snail, Twist, ZEB1), as well as epithelial (E-cadherin, GRHL2 and EpCAM) and mesenchymal (N-cadherin and Vimentin) markers, (2) determine the relationship between EMT and clinicopathological parameters such as histological type and grade, molecular subtype, and onset of metastases, (3) evaluate the nature and intensity of the immune response using a semiquantitative assessment of the inflammatory infiltrate present in breast cancer, and (4) ascertain the impact of each EMT-related marker on the inflammatory cell response.

In the present study, we showed that the inflammatory infiltrate was more developed in invasive ductal carcinoma and was more often found to be associated with poorly differentiated breast cancer including triple-negative carcinomas. The disruption of the protein expression of Vimentin and specific epithelial markers markedly influenced the magnitude of the inflammatory infiltrate found in the TME. Most of the studied EMT-related markers point to a plasma cell infiltration of the TME.

## 2. Materials and Methods

### 2.1. Patients and Tissue Samples

A total of 144 consecutive cases, with primary invasive breast cancer, who underwent mastectomy at the Hôtel Dieu Hospital and at the Centre Hospitalier de l’Université de Montréal (CHUM) between 2001 and 2018 were included in the present study. Tissue microarrays (TMA) were constructed using representative formalin-fixed paraffin-embedded (FFPE) tumor blocks of these cases, as previously described [27]. Clinical data of patients and tumor characteristics were retrieved from the patients’ medical records and pathological reports. The histological grade was confirmed using Ellis’s Modified Scarff–Bloom–Richardson–Elston grading system [28]. The immuno-histochemical staining of the surrogate markers ER, PR, HER2, and Ki-67 was used to classify breast cancer tumors into the different molecular subtypes: luminal A, luminal B, HER2-positive, and TNBC [29,30]. The study was approved by the CHUM ethics committee (SL 05-019), and all data were retrospectively analyzed.

### 2.2. Assessment of the Inflammatory Infiltrate in the Tissue Samples

The inflammatory infiltrate was evaluated according to the guidelines of the International Immuno-Oncology Biomarker Working Group (2015) [31]. The overall assessment was based on the semiquantitative measurement of stromal inflammatory infiltrate on 4 μmthick H&E-stained whole histological sections that were scanned at 40× magnification (Nanozoomer^®^ Digital Pathology; Hamamatsu Photonics K.K., Hamamatsu City, Japan). For each section, the entire surface was evaluated to measure the intensity of the inflammatory infiltrate expressed as a percentage whose denominator is the surface of stromal tissue, and whose nominator is the surface occupied by the inflammatory cells. Two groups were dichotomously defined on the basis of the immune infiltrate: mild (<10%) or intense (≥10%). The following regions were excluded: necrotic/hemorrhagic regions, those containing artefacts, infiltrate surrounding benign structures, normal lobules, and regions containing regressive areas of fibrosis or hyalinization.

### 2.3. Characterization of the Inflammatory Infiltrate in the TME

The characterization of the inflammatory infiltrate was carried out using immunohistochemistry-labeled tissue microarrays. We evaluated the presence of CD3^+^ T lymphocytes, CD20^+^ B lymphocytes, and MUM1^+^ plasma cells. The immune infiltrate was considered mild if the sum of the percentages of cells stained by these threemarkers was less than 10%. In turn, it was considered intense if the sum was higher or equal to 10% [32]. At the same time, we assessed subsets of tumor-infiltrating lymphocytes (TILs) including CD4^+^ helper T lymphocytes, CD8^+^ cytotoxic T lymphocytes, and FOXP3^+^ regulatory T lymphocytes (Tregs). Double immunohistochemical labeling for CD4/CD8, FOXP3/CD4, and CD20/CD3 was performed to evaluate the nature of the inflammatory infiltrate present in the stroma of breast cancer and establish correlation with EMT-related markers included in this study.

### 2.4. Tissue Microarray

Sections (4 μm) from each paraffin donor block were stained with H&E, and a representative tumor area was identified. Duplicate or triplicate core punches, 1 mm in diameter, were plucked from representative areas contained within each FFPE tumor block. The cores were realigned into recipient blocks, according to the intended design of the map, using a Manual Tissue Arrayer I (Beecher Instruments; Estigen OÜ, Tartu, Estonia). Blocks were next inverted and incubated overnight in the oven at 40 °C over a glass slide. TMA blocks were allowed to cool until they could easily detach from the glass slide. Tissue sections from each TMA were prepared, and one slide from the block was stained with H&E to review the diagnosis and confirm histological grades on all tissue samples. Additional representative sections from each block were submitted to immunohistochemical (IHC) staining [27].

### 2.5. Immunohistochemistry

To assess the level of protein expression of the EMT-related markers and characterize the immune infiltrate for each case of the cohort, IHC reactions for Snail, Twist, ZEB1, GRHL2, E-cadherin, N-cadherin, Vimentin, EpCAM, CD3, CD4, CD8, CD20, FOXP3, and MUM1, as well as β-catenin, were carried out on 4 μmthick histological sections of the TMAs described above. IHC reactions were performed on the Bond RX Stainer (Leica Biosystems, Buffalo Grove, IL, USA) and the BenchMark ULTRA IHC/ISH System (Ventana Medical Systems, AZ, USA) according to the manufacturers’ instructions. The antibodies, clones, dilutions, pretreatment, and treatment conditions, as well as the suppliers, are listed in Table 1.

### 2.6. Evaluation of the Immunohistochemical Labeling

A semiquantitative evaluation of the expression of each marker by IHC was carriedout. For Snail and ZEB1 labeling, only nuclear staining was considered. The reaction was considered positive when more than 10% of the neoplastic nuclei were labeled; the reaction was considered negative otherwise [33]. The 10% cutoff was also applied for N-cadherin; when more than 10% of the tumor cells displayed a circumferential membrane labeling, the reaction was considered positive. The absence of membrane labeling or labeling (continuous or discontinuous) occurring in less than 10% of tumor cells was considered negative [33]. In order to evaluate E-cadherin, we relied on the loss of protein expression. When compared to a positive internal or external control, the level of E-cadherin expression was sorted out into three categories on the basis of the intensity of the staining and the percentage of tumor cells labeled. Thus, the reaction was deemed (1) positive if the circumferential membrane staining present in the tumor cells was comparable to normal tissue or positive control tissue, (2) reduced if circumferential membrane labeling in tumor cells failed to reach that observed in normal tissue (or positive control) and/or if the labeling was heterogeneous, incomplete, or absent in more than 10% of the cells, and (3) negative when there was a complete absence of membrane labeling [34].The evaluation of Twist, EpCAM, and GRHL2 labeling was performed using the Histo-score (H-score), which includes a range of scores varying from 0 to 300. This score was based on the evaluation of two parameters: the percentage of cells labeled and intensity of the reaction. The level of expression of the two markers was ascribed to one or the other of two groups: negative (low) if the score was lower than 100 and positive (high) if the score was greater than 100 [35]. As for Vimentin, a semiquantitative evaluation of the cytoplasmic labeling was used. Briefly, cases were classified into four groups: negative, i.e., no cytoplasmic labeling; 1+, less than 10% labeled tumor cells; 2+, 10–50% labeled tumor cells; 3+, more than 50% of tumor cells with cytoplasmic labeling. Vimentin expression level was considered to be positive in groups 1+, 2+, or 3+ [36].The level of expression of β-catenin was sorted into four grades according to the intensity of the staining; (1) negative: absence of staining; (2) membranous: circumferential membrane staining present in the tumor cells; grade 1: loss of membranous staining; grade 2: cytoplasmic expression with or without loss of membranous staining [37,38].

Double labeling was used to establish the CD4/CD8, FOXP3/CD4, and CD20/CD3 ratios. The CD4/CD8 ratio is the number of cells that show membrane expression of CD4 divided by the number of cells that show membrane expression of CD8. The FOXP3/CD4 ratio corresponds to the number of cells which display both membrane expression of CD4 and nuclear expression of FOXP3 divided by the total number of CD4-positive cells. The CD20/CD3 ratio is based on the number of cells that show membrane expression of CD20 divided by the number of CD3-positive cells. MUM1 was used to determine the percentage of plasma cells infiltrating the tumor stroma. It corresponds to the ratio of the area occupied by MUM1-positive cells relative to the total area of stroma reported as a percentage. The same principle generally applied for the assessment of CD3 and CD20.

### 2.7. Statistical Analysis

Statistical analysis was performed using SPSS software, version 25. The χ^2^ (chi-square) test was used to assess the correlation between clinical–pathological data and studied parameters such as the level of expression of EMT-related markers and the degree of inflammatory infiltrate, as well as the association between these same parameters. In order to assess the correlation between the level of expression of EMT-associated markers and the different cell ratios described above, a comparison of the medians was selected using IBM SPSS statistical software.

## 3. Results

### 3.1. Clinicopathological Characteristics of the Cohort

As a first step, we determined the clinical–pathological characteristics of the cohort, which included 144 breast cancer cases. Most of these cases were diagnosed as infiltrating ductal carcinomas (87.5%), while fewer cases were classified as invasive lobular carcinomas (9.7%) or undifferentiated medullary-like carcinomas (2.8%). Half of the cohort (56.2%) consisted of well to moderately differentiated carcinomas (grade I and II), while the remainder (43.8%) were poorly differentiated (grade III). The evaluation of molecular subtypes indicated that luminal A or B subtype predominated (63.2%), followed by TNBC (29.9%). The HER2-positive subtype accounted for only 6.9% of cases. Only one-third (29.2%) of cases developed lymph node metastases, while the remainder (67.3%) were node-negative (Table 2).

### 3.2. Association between the Levels of Expression of EMT-Related Markers and the Clinicopathological Criteria

To correlate the protein expression of the selected EMT-related markers and the various clinical–pathological criteria, the level of expression of these markers was next evaluated by IHC (Supplementary Figures S1 and S2I,II).

### 3.3. The Mesenchymal Markers N-Cadherin and Vimentin Were Associated with the Histological Grade and the Molecular Subtype

**Figure 1:**
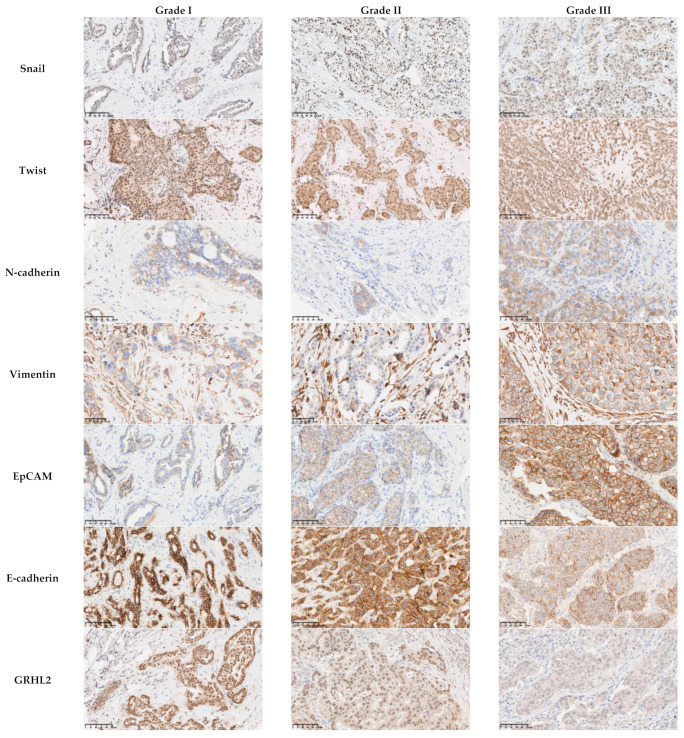
Association between the protein expression of EMT-related markers and the histological grade.There was no association between the level of expression of the mesenchymal markers Snail (*p* = 0.149) and Twist *(p* = 0.38) and the histological grade. The mesenchymal markers N-cadherin and Vimentin and the epithelial marker EpCAM were overexpressed in grade III carcinomas (*p* = 0.033, *p* < 0.0001, and *p* < 0.001, respectively). The reduction in the expression of E-cadherin was more often observed in grade III carcinomas (*p* = 0.001). All the cases with a low or negative expression level of GRHL2 were grade III (*p* = 0.001). Scale bars: 100 μm.

**Figure 2:**
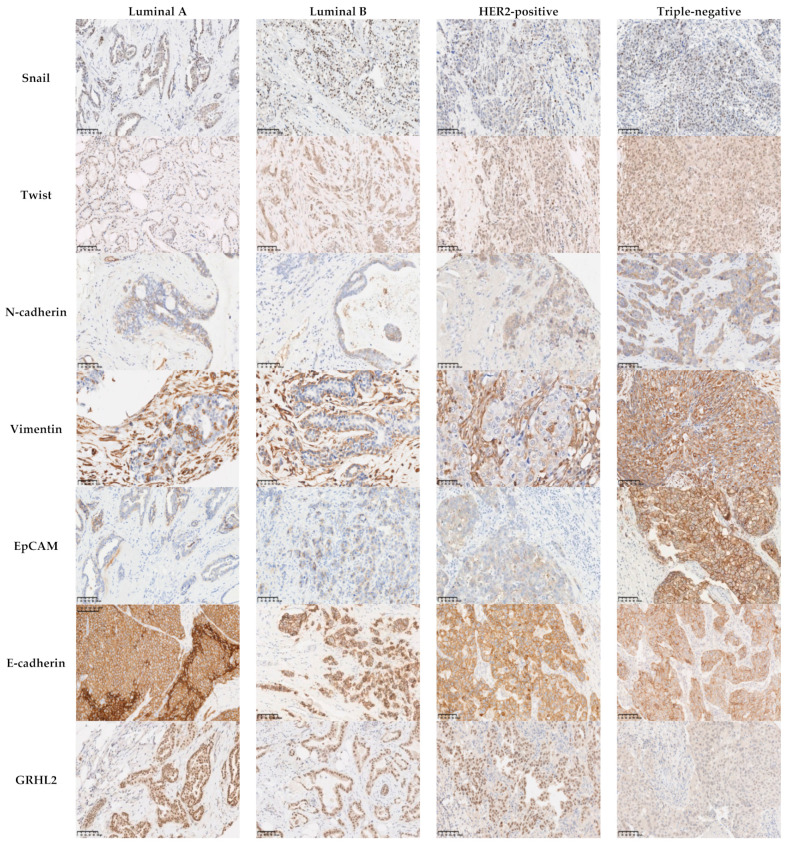
Association between the protein expression of EMT-related markers and breast cancer molecular subtypes. There was no association between the expression of the mesenchymal markers Snail (*p* = 0.176) and Twist (*p* = 0.617) and the molecular subtypes. The mesenchymal markers N-cadherin and Vimentin and the epithelial marker EpCAM were overexpressed in triple-negative carcinoma (*p* = 0.001, *p* < 0.0001, and *p* < 0.001, respectively). Areduction in the levels of expression of E-cadherin was more often observed in triple-negative carcinomas (60.5%; *p* = 0.001), while 81% of the cases with a low or negative expression levels of GRHL2 were triple-negative (*p* < 0.001). Scale bars: 100 μm.

Analysis of the IHC results showed that there was no significant relationship between the level of expression of the mesenchymal markers Snail and Twist and the histological subtype, histological grade, molecular subtype, or lymph node metastasis (*p* > 0.05). On the contrary, the levels of expression of N-cadherin and Vimentin were associated with the histological grade and molecular subtype. In fact, N-cadherin and Vimentin were found to be overexpressed in grade III carcinomas (*p* = 0.033 and *p* < 0.0001, respectively) and the triple-negative subtype (*p* = 0.001 and *p* < 0.0001, respectively) (Table 3, Figure 1 and Figure 2). Moreover, the level of expression of Vimentin was associated with the histological subtype (*p* = 0.048). ZEB1 was detected in only one case of the cohort (Supplementary Figure S3).

### 3.4. The Epithelial Markers E-Cadherin, GRHL2, and EpCAM Were Associated with the Histological Grade and the Molecular Subtype

Analysis of the association between the level of protein expression of the epithelial markers and the clinicopathological parameters showed findings worthy of note. The reduction in the expression of E-cadherin was more often observed in grade III carcinomas and triple-negative carcinomas (54% and 60.5%, respectively; *p* = 0.001). All cases with a low or negative expression level of GRHL2 were grade III (*p* = 0.001), and nine out of eleven (81%) were triple-negative (*p* < 0.001). EpCAM was found to be overexpressed in grade III and triple-negative carcinoma with values of 54% and 66.7%, respectively (*p* < 0.001). Interestingly, our results showed a negative association of EpCAM expression with lymph node metastases (*p* = 0.02), which underscores the intriguing role of EpCAM in lymph node metastases (Table 4; Figure 1 and Figure 2).

### 3.5. Poorly Differentiated, HER2-Positive and Triple-Negative Carcinomas Were More Often Associated with Inflammatory Infiltrate

**Figure 3:**
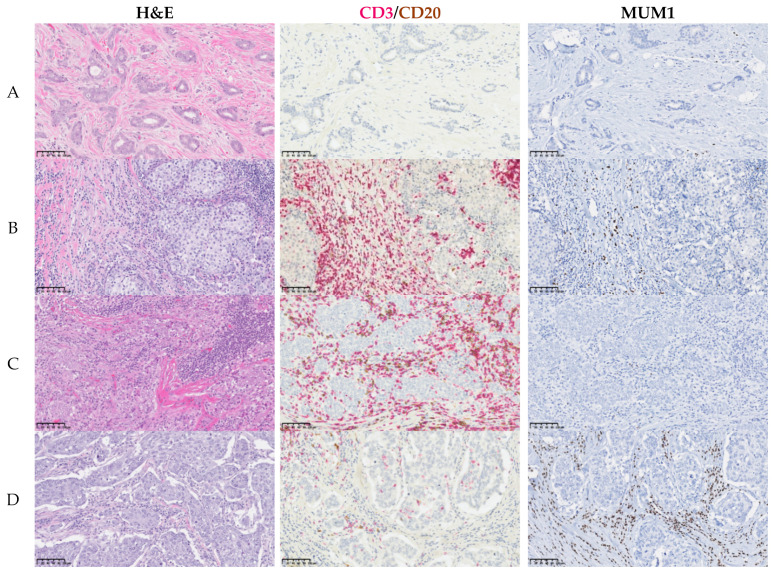
Inflammatory infiltrate in breast cancer. Representative images of fourcases showing whole H&E-stained sections and IHC (single or double) assessment of immune cells on TMAs for the most abundant immune populations CD3 (red)/CD20(brown) and MUM1. (**A**) Mild infiltrate; (**B**) intense infiltrate with CD3^high^, CD20^low^, and MUM1^low^ expression; (**C**) intense infiltrate with CD3^high^, CD20^high^, and MUM1^low^ expression; (**D**) intense infiltrate with CD3^low^, CD20^low^, and MUM1^high^ expression. Scale bars: 100 μm.

Assessment of the inflammatory infiltrate in tumor tissue samples was next performed to determine the percentage of infiltration and to classify cases into two categories, mild (<10%) or intense (≥10%) inflammatory infiltrate, as previously described. This was followed by studying the association between the clinical–pathological criteria and the degree of inflammatory infiltrate evaluated on whole H&E-labeled sections and IHC-labeled TMAs (Figure 3). The number of available cases to be evaluated by IHC was less than thatavailable for evaluation by H&E due to fragmented or absent cores in the TMAs; however, our findings showed consistency and comparable statistical significance. Our results showed that the inflammatory infiltrate was more pronounced in invasive ductal carcinoma than in invasive lobular carcinoma (*p* = 0.013 and *p* = 0.02, respectively), where only one case showed an intense infiltrate. Likewise, poorly differentiated carcinomas, HER2-positive carcinomas, and triple-negative carcinomas were more often associated with a brisk inflammatory infiltrate detected by H&E (41.3%, 60%, and 41.9%, respectively; *p* < 0.0001), as well as by IHC (49.1%, 66.7%, and 47.4%, respectively; *p* < 0.0001) (Table 5).

### 3.6. The Expression of Epithelial Markers Related to EMT Was Associated with the Degree of Inflammatory Infiltrate

To explore the potential role of EMT as it relates to immune infiltration of the tumor microenvironment, we studied whether the levels of protein expression of the mesenchymal and epithelial EMT-related markers were associated with the extent of the inflammatory infiltrate. Our results indicated that there was no significant association between the mesenchymal markers and the degree of inflammatory infiltrate (*p* > 0.05) except for Vimentin (Table 6). Cases with overexpression of Vimentin (42.9%) were associated with an intense inflammatory infiltrate present on IHC-stained TMAs (*p* = 0.004); Regrettably, the statistical significance could not be maintained upon studying the association with the degree of inflammatory infiltrate measured on H&E-stained whole sections (*p* = 0.052). With regard to the epithelial markers, our findings showed interesting results. In particular, cases with a reduced level of expression of E-cadherin were associated with intense inflammatory infiltrate detected on both H&E- and IHC-labeled sections (*p* = 0.005 and *p* = 0.03, respectively). Likewise, cases where the GRHL2 wasreduced or abolished were associated with intense inflammatory infiltrate evaluated on both H&E- and IHC-labeled sections (*p* = 0.013 and *p* = 0.001, respectively). In contrast, cases with overexpression of EpCAM were associated with an intense inflammatory infiltrate assessed by both H&E and IHC (*p* = 0.037 and *p* < 0.0001, respectively) (Table 6).

### 3.7. Association between the EMT-Related Markers and the Different Cells Which Constitute the Immune Infiltrate of the TME

Next, we wanted to study the association between the seven markers of EMT and TIL subsets which constitute the immune infiltrate of the TME. The comparison of the medians for the CD4/CD8, FOXP3/CD4, and CD20/CD3 ratios showed interesting results with regard to both N-cadherin and EpCAM. Cases that expressed N-cadherin weregenerally more infiltrated with CD4 Helper T cells (*p* = 0.004) than negative cases. On the other hand, cases that expressed EpCAM were more infiltrated with B lymphocytes (CD20) than cases that failed to express this marker (*p* = 0.03). Otherwise, no significant differences could be found between the groups expressing and not expressing the markers related to EMT.

**Figure 4:**
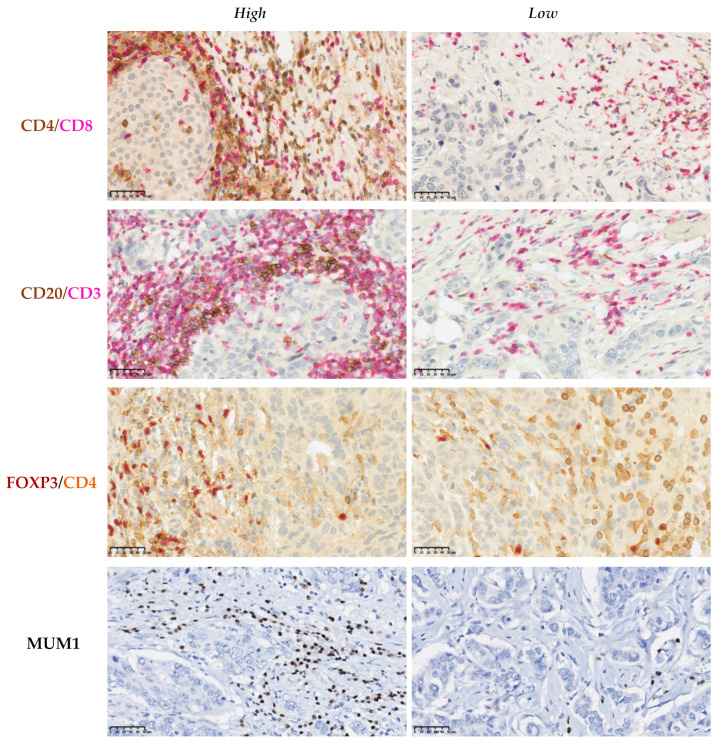
Immunohistochemical assessment of the immune cells in the tumor microenvironment of breast cancer. Double IHC labeling was used to establish the *CD4/CD8*, *CD20/CD3*, and *FOXP3/CD4* ratios. Representative sections showing *high* and *low* ratios of these markers. IHC labeling with MUM1 was used to determine the percentage of plasma cells that infiltrate the tumor stroma. Scale bars: 50 μm.

**Figure 5:**
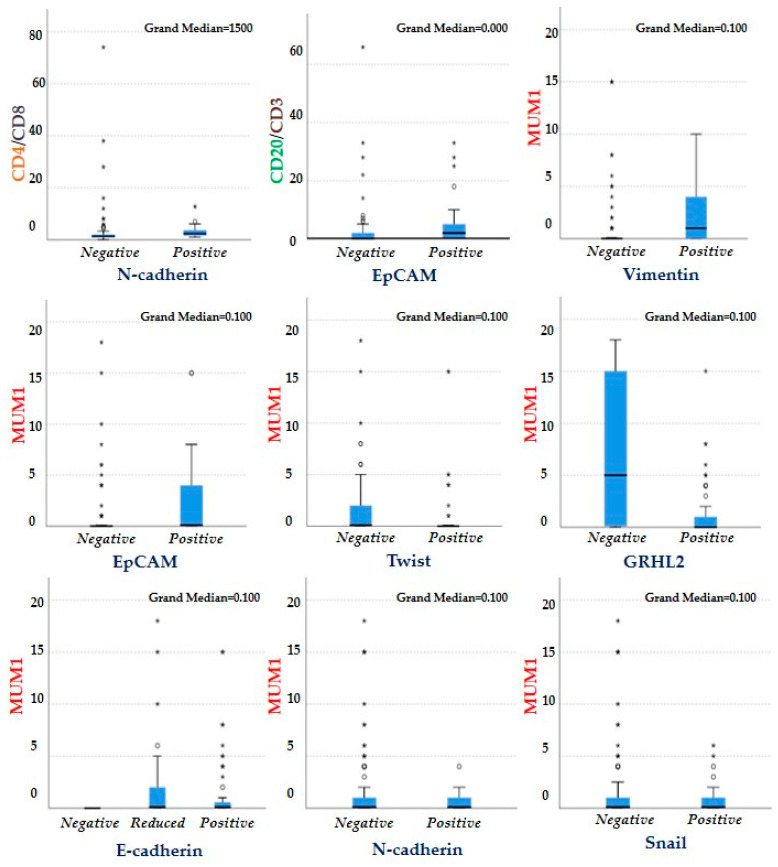
Association between the EMT-related markers and markers of immune cells present in the TME using the independent samples median test. Cases that expressed N-cadherin were generally more infiltrated with CD4 helper T cells (*p* = 0.004) than negative cases, while cases that expressed EpCAM were more infiltrated with B lymphocytes (CD20) than cases that failed to express this marker (*p* = 0.03). Cases that expressed Vimentin and EpCAM were more infiltrated with plasma cells than those that did not express them (*p* = 0.001, *p* = 0.008, respectively), where as cases that expressed Twist and GRHL2 were less infiltrated by plasma cells (*p* = 0.039 and *p* = 0.03, respectively). Cases that did not express E-cadherin were not infiltrated by plasma cells (*p* = 0.005). *,°: outliers.

Comparison of the medians for MUM1 showed that there was an association between the level of expression of Twist, Vimentin, E-cadherin, EpCAM, and GRHL2 and the percentage of plasma cells in the TME. The cases that expressed Twist and GRHL2 wereless infiltrated by plasma cells (*p* = 0.039, *p =* 0.03, respectively). The cases that expressed Vimentin and EpCAM were more infiltrated by plasma cells than the cases that did not express them (*p* = 0.001, *p* = 0.008, respectively). Our data indicate that E-cadherin negative lobular carcinoma subtype was not infiltrated by plasma cells (*p* = 0.005) (Table 7 and Figure 4 and Figure 5).

### 3.8. Association between the Combined Epithelial and Mesenchymal Markers and the Inflammatory Infiltrate

Next, we stratified the cases in our cohort into four groups on the basis of the expression patterns of E-cadherin and the fourmesenchymal markers (Snail, Twist, N-cadherin, and Vimentin) as shown in Table 8. Group 1 represents cases whose tumors remained in a largely epithelial state expressing only E-cadherin. Group 2, representing a hybrid phenotype, was the group strongly associated with an intense inflammatory infiltrate (34.7%). In contrast, all cases in Group 3, representing a lobular carcinoma phenotype, showed a mild inflammatory infiltrate (*p* = 0.03). However, none of these four groups were associated with specific TILs subsets CD4/CD8 (*p* = 0.2) and FOXP3/CD4 (*p* = 0.31).

### 3.9. The Staining Patterns of E-Cadherin and β-Catenin Are Associated and Positively Correlated

Given the complex interaction between β-catenin and E-cadherin, we wanted to establish if there was an association between the four staining patterns of β-catenin and the three staining patterns of E-cadherin. A positive correlation could be established between these two markers (*p* < 0.0001), as shown in Table 9. All cases with E-cadherin-negative labeling were also negative for β-catenin. As expected, E-cadherin-positive labeling was associated with membranous β-catenin labeling. Furthermore, 51.2% of the cases with reduced E-cadherin staining displayed reduced β-catenin labeling (Grade 1) (*p* = 0.02) (Supplementary Figure S4). Regrettably, we were unable to investigate the association between N-cadherin and β-catenin in our studied cohort due to the small number of cases presenting with a positive N-cadherin expression pattern.

## 4. Discussion

Tumor immune landscapes vary tremendously in breast cancer, and it is of great importance to study how the drivers of tumorigenesis interact to modulate the tumor immune milieu [26]. Breast cancer is a highly complex and heterogenous disease, with several molecular subtypes and even sub-subtypes; thus, understanding the impact of breast cancer EMT signature on reprogramming the TME may enable personalized immune intervention modalities.

The evaluation of the inflammatory infiltrate of the TME appears to hold promise in terms of prognostic and predictive values in breast cancer [39,40,41,42]. Distinct molecular subtypes of human breast cancer can be associated with a defined immune profile and activation status in the TME [26]. According to van Rooijen et al., triple-negative carcinomas, which are generally poorly differentiated, exhibit high genomic instability that increases the mutational load and the level of expression of tumor neo-antigens. This results in the development of a strong immune response [43]. In contrast, tumors harboring the estrogen receptor have been shown to promote a T helper2 (Th2) pro-tumorigenic immune environment and to downregulate MHC class II expression in breast cancer cells [44,45], thus leading to a weaker immune response. Our results clearly indicated that the lobular subtype was much less infiltrated than the ductal type. These results could be explained by the predominance of the ductal subtype (87.3%) in our cohort and the fact that the lobular subtype is generally well differentiated.

Among our studied cases, the tumors, representing a hybrid phenotype, were highly associated with intense immune infiltrate. A recent study elucidated that, in breast cancer, the hybrid phenotype can be endowed with a highly immune-evasive character through increased PD-L1 levels [46]. Tumors can facilitate the accumulation of immune-suppressive cells, inhibit the function of effector T cells, or induce a population of tolerogenic cells that result in immune escape of the tumor [47].

During tumor progression, cancer cells and the different TME cell types influence each other to modulate the cell–cell junctions between cancer cells and those between TME cell types and cancer cells [48].Our findings provide some original insights regarding the impact of the EMT-related markers on theoverall and the cell-specific composition of the immune infiltrate in the TME of breast cancer. Although both the cellular and humoral arms of the immune system are involved in tumor development and progression [49], herein we showed a strong association between most of the studied EMT markers and tumor-infiltrating plasma cells. EpCAM expression was positively associated with TNBC and with infiltration of the TME by MUM1^+^ plasma cells and CD20^+^ TILs. Likewise, downregulation of GRHL2 and upregulation of Vimentin were associated with plasma cell infiltration and TNBC. These findings are in line with previous reports indicating the significant elevation of CD20^+^ TILs in TNBC compared to other molecular subtypes [50]. Furthermore, Bar et al. demonstrated the overexpression of miR-210 in MUM1^+^ immunoglobulin-producing tumor-infiltrating plasma cells in TNBC [51], which suggests that tumor cells might modulate miR-210 expression to influence plasma cell function. Alternatively, miR-210 overexpression might stem from enhanced activation of plasma cells in the tumor stroma, leading to increased immunoglobulin production [51].

Lobular carcinoma cases, which are typically E-cadherin-negative, were devoid of plasma cells infiltration. Interestingly, the epithelial phenotype, represented by Twist downregulation, was associated with plasma cell infiltration of the TME. Plasma cells or B lymphocytes could assume pro-tumoral or anti-tumoral roles under certain conditions; however, the factors driving the emergence of these phenotypes and the roles played by plasma cells and B lymphocytes in these circumstances have yet to be unraveled [49]. At any rate, the predictive and prognostic values of plasma cell infiltration in breast cancer remains a challenging issue [49,52,53].

Given the success of monoclonal antibody-based immunotherapy, these results might pave the road to the development of new interventions that are capable of exploiting the humoral immune response in breast cancer [54].

The association between N-cadherin upregulation and CD4^+^ and CD8^+^ T cells is intriguing. CD4^+^ T cells, either on their own or by cooperating with other immune cells, constitute critical determinants of effective antitumor immune responses. The distinct CD4^+^ T-cell subsets have diverse impact on tumor growth [55]. CD4^+^ T helper 1 (Th1) cells play a role in protecting against tumor growth, whereas CD4^+^ Th2 [56] and CD4^+^ FOXP3^+^ Treg cells promote tumor growth [57]. Cytotoxic CD8^+^ lymphocytes have been considered the main mediators of the immune surveillance directed against tumors [58]. We assume that these tumors might have been infiltrated by CD4^+^ Th2 TILs and less infiltrated by CD8^+^ lymphocytes since N-cadherin was associated with poorly differentiated tumors; however, this hypothesis should be investigated in future studies. Chen et al. demonstrated a molecular link between EMT and CD8^+^ TIL immunosuppression via the regulation of PD-L1 in both human cell lines and animal models, thus creating a suppressive TME [59].

Understanding the strategies of tumor-driven reprogramming of the TME would be a major step toward more successful guidance of different therapeutic modalities [46]. In inflammatory breast cancer, which is a rare and aggressive type of breast cancer, a number of pathways such as the *JAK/STAT*, *COX2*, and *IL6* pathways are activated, which have critical biological effects by supporting the escape of cancer cells from detection by the immune cells in the TME [48].

For the moment, the molecular mechanisms linking the markers of EMT and the immune system are still poorly understood. Most of the studies performed to date have been conducted in animal models involving the implantation of tumors developed from cell lines in which mesenchymal markers have been overexpressed. The tumors that have arisen are sometimes epithelial and sometimes mesenchymal. Notably, it has been observed that epithelial tumors are generally more infiltrated than mesenchymal tumors [17].

Our findings revealed that, using a semiquantitative assessment, our cohort could not establish a firm correlation between the level of protein expression of Snail, Twist, or N-cadherin and the degree of inflammatory infiltrate. On the contrary, there was a significant correlation between the level of expression of Vimentin and specific epithelial markers with the degree of inflammatory infiltrate. It has been suggested that the loss of E-cadherin precedes the gain of mesenchymal markers in most tumor cells exhibiting morphological features of EMT, thus facilitating their identification by the loss of E-cadherin staining rather than by a gain of any single mesenchymal marker [60].

The reduction in the expression of E-cadherin, which can be viewed as a hybrid EMT state [61], was significantly associated with immune infiltrate of the TME. Of note, our results are concordant with those of a previous study demonstrating an inverse correlation between the inflammatory infiltrate and E-cadherin protein expression in gall bladder cancer [62].

The phenomenon of EMT manifests itself by the coordinated recruitment of different genes and signaling pathways in each of the molecular subtypes of breast cancer. Even though several studies have shown that overexpression of Snail, Twist, and N-cadherin leads to aggressive cancer progression [18,63,64,65], our findings demonstrated that the levels of protein expression of the transcription factors Snail and Twist failed to hold significant association with the studied clinical–pathological criteria. N-cadherin, a recognized marker of mesenchymal differentiation and strongly linked to cell mobility, was correlated with the histological grade and molecular subtype. A conceivable mechanism is that N-cadherin binds β-catenin molecules to stabilize cell adhesion. The overexpression of N-cadherin ensures a pool of β-catenin for the functioning of the Wnt pathway, which, once activated, induces the dissociation of the N-cadherin/β-catenin complex. The β-catenin molecules can then translocate to the nucleus to activate the transcription of genes stimulating cell division and growth [18]. Vimentin regulates cell migration via inhibition of focal adhesion-associated proteins and contributes to angiogenesis through its role in Notch signal transduction and induction of vasculogenic mimicry [25]. ZEB1 was expressed by tumor cells from a single case, in our studied cohort, where the tumor stroma indicated the presence of a positive nuclear staining for ZEB1. However, such EMT cells in the stroma are extremely difficult if not rightly impossible to distinguish from fibroblasts which naturally express ZEB1.

E-cadherin has shown a significant association with most of the clinicopathological parameters except for lymph node metastasis. A plausible mechanism is that E-cadherin helps stabilize cell junctions and interacts via its cytoplasmic domain with β-catenin, where it also competes with the Wnt signaling pathway. Since β-catenins are associated with proto-oncogenes, they confer tumor-suppressing properties on E-cadherin. The reduction in or even the loss of E-cadherin releases β-catenins into the cytoplasm, thereby activating the Wnt signaling pathway to promote their passage to the nucleus [66]. Since the loss of E-cadherin is a hallmark of lobular carcinoma [67], the correlation observed with the histological subtypes appears to be significant. Interestingly, we demonstrated a positive correlation between the levels of protein expression of E-cadherin and β-catenin, which adequately supports the findings of previous reports [37,68].

GRHL2 plays a crucial role in the maintenance of the epithelial phenotype as it functions as a regulator of the expression of cadherins [69]. Its direct interaction with E-cadherin may help explain how suppression of GRHL2 promotes the aggressiveness of tumors. These results support and expand previous observations showing that a complete EMT signature with concomitant loss of epithelial markers was identified in the basal-like subtype of breast cancer cell lines, while a partial EMT signature prevailed in luminal A/B subtypes [70]. EpCAM expression showed a strong association with all the clinicopathological parameters. EpCAM is known to stimulate the transcription and translocation of c-myc via its intracytoplasmic domain which, after proteolytic cleavage, leads to cell proliferation [24,71]. The negative association between EpCAM and E-cadherin expression is intriguing; in vitro studies have shown that EpCAM negatively modulates E-cadherin-mediated adhesions and decreases its cytoskeleton–bound fractions via disrupting the link between F-actin and α-catenin [72]. Another proposed mechanism is via activation of the Wnt pathway, which depends on the availability of cytoplasmic α-catenin [73]. Consequently, EpCAM silencing significantly reduces the availability ofcytoplasmic α-catenin, by increasing its association with E-cadherin, thusabolishing those molecules needed for the Wnt pathway and, hence, shutting down the activation of its target genes [74]. This negative correlation may explain the link between EpCAM overexpression with tumor aggressiveness [75]. However, one would be hard-pressed to explain how lymph node metastasis is inversely associated with the expression of EpCAM. Abrogation of *EPCAM* has been associated with invasion and migration in MCF10A cells [76]. Furthermore, *EPCAM* knockdown in esophageal and head and neck carcinoma cells resulted in an increase in their migratory capacity following Vimentin expression [77]. This study indicates that progressive loss of membranous EpCAM with the appearance of cytoplasmic positive speckles provides evidence of EpCAM endocytosis and degradation [77], which was more often observed in migrating and invading cells. In esophageal cancer, whereas EpCAM^high^ phenotypes are associated with proliferative stages during the initial growth of the tumor, EpCAM^low/negative^ phenotypes are correlated with migration, invasion, and dissemination [77].

The present study has several strengths: the assessment of eight EMT-related markers on tissue samples available from 144 breast cancer patients, as well as a prospectively defined hypotheses and analysis plan. To our knowledge, this is the first study evaluating the impact of EMT-related markers on the inflammatory infiltrate in the TME of breast cancer using patients’ tissue samples. We also assessed the overall immune infiltrate on H&E-stained whole sections and characterized the immune populations on IHC-stained TMAs using sixmarkers. Lastly, we minimized the difference between the two studied methods by evaluating multiple TMA cores widely distributed across the tumor complemented with whole sections of the tumors.

However, our study is not without limitations. Although we selected eight representative EMT-related markers, there are several other known EMT-related markers that would have been interesting to evaluate in this context. Furthermore, a highly challenging obstacle concerns the positive identification and distinction of tumor cells undergoing EMT from stromal fibroblasts by IHC. Arguably, it is possible that we underestimated the extent of labeled tumor cells, especially considering a marker like ZEB1. Evaluations on TMAs have their own limitations; however, studies analyzing whole sections spanning the center and margins of primary tumors from invasive breast cancer have shown highly concordant IHC expression levels of EMT markers [78,79], which suggest that no change in our results would have been obtained from the analysis of full-face tissue sections.

## 5. Conclusions

To conclude, the results presented herein show that deregulation of protein expression of both epithelial markers and vimentin is significantly correlated with the nature of the inflammatory infiltrate. Each breast cancer molecular subtype showed distinct patterns of expression of EMT-related markers, as well as varying degrees of plasma cell infiltration. Taken together, this suggests that the crosstalk across these genes, signaling pathways, and the immune cells bear significant impact on the immune response mounted in the TME and, hence, the aggressiveness of the tumor. The molecular mechanisms via which these markers function in the TME are not yet fully understood and will require further investigation. Nevertheless, these novel findings clearly identify some of the underlying tumor characteristics which dictate the inter-tumor heterogeneity. A more complete characterization of immune landscapes may open new avenues for rational decision making in the clinical use of immunotherapy in subsets of breast cancer patients, especially TNBC.

## Figures and Tables

**Table 1:** List of antibodies used in the immunohistochemical staining.

Antibody	Source	Clone	Dilution	Unmasking	Incubation (min) *	Detection	Location of the Staining
Snail (AMAb91215)	Atlas antibody Bromma, Sweden	CL3700	1/3000	Citrate	30/15/15	Peroxidase polymer (HRP-DAB)	Nuclear
Twist (ab50581)	Abcam Cambridge, UK		1/1000	Citrate	15/0/8	Peroxidase polymer (HRP-DAB)	Nuclear
ZEB1 (ab180905)	Abcam Cambridge, UK	OTI3G6	1/1000	Citrate	15/8/8	Peroxidase polymer (HRP-DAB)	Nuclear
E-cadherin (M3612)	DAKO Agilent Santa Clara, CA, USA	NCH-38	1/50	EDTA	60/30/30	Peroxidase polymer (HRP-DAB)	Membrane
N-cadherin (ab225719)	Abcam Cambridge, UK	SP90	1/50	EDTA	30/0/15	Peroxidase polymer (HRP-DAB)	Membrane
EpCAM (ab223582)	Abcam Cambridge, UK	EPR20532-225	1/500	EDTA	30/0/15	Peroxidase polymer (HRP-DAB)	Membrane
GRHL2	Sigma-Aldrich St. Louis, MO, USA	HPA004820	1/100	EDTA	30/0/15	Peroxidase polymer (HRP-DAB)	Nuclear
Vimentin Bs-0756R	Bioss Antibodies Inc. Woburn, MA, USA		1/500	Citrate	30/0/15	Peroxidase polymer (HRP-DAB)	Cytoplasm
β-catenin	Ventana Medical Systems, Roche Diagnostics, Canada	14	RTU	EDTA	24/28	Peroxidase polymer (HRP-DAB)	Membrane
CD3 (PA0553)	Leica Biosystems, Newcastle, UK	LN10	BOND RTU	EDTA	Protocol F	Red polymer (AP-Fast Red)	Membrane
CD4 (M7310)	DAKO Agilent Santa Clara, CA, USA	4B12	1/50	EDTA	30/15/15	Peroxidase polymer (HRP-DAB)	Membrane
CD8 (GA623)	DAKO Agilent Santa Clara, CA, USA	C8/144B	1/200	EDTA	30/15/15	Red polymer (AP-Fast Red)	Membrane
CD20 (PA0200)	Leica Biosystems, Newcastle, UK	L26	BOND RTU	Citrate		Peroxidase polymer (HRP-DAB)	Membrane
FoxP3 (ab20034)	Abcam Cambridge, UK	236A/E7	1/100	EDTA	30/15/15	Red polymer (AP-Fast Red)	Nuclear
MUM1 (GA644)	DAKO Agilent Santa Clara, CA, USA	MUM1p	1/200	EDTA	48/32	Peroxidase polymer (HRP-DAB)	Nuclear

* Incubation primary antibody/secondary antibody/polymer; RTU: ready to use; HRP: horseradish peroxidase; DAB: 3,3′-Diaminobenzidine tetrahydrochloride hydrate; AP: alkaline phosphatase.

**Table 2:** The clinicopathological characteristics of 144 breast cancer patients.

Clinicopathological Characteristics	Number (Percentage)
Histological subtype	
Infiltrating ductal carcinoma (IDC)	126 (87.5%)
Infiltrating lobular carcinoma (ILC)	14 (9.7%)
Undifferentiated medullary-like carcinoma	4 (2.8%)
Histological grade	
Grade I	31 (21.5%)
Grade II	50 (34.7%)
Grade III	63 (43.8%)
Molecular subtype	
Luminal (A/B)	91 (63.2%)
HER2-positive	10 (6.9%)
Triple-negative	43 (29.9%)
Lymph node metastases	
Yes	42 (29.2%)
No	97 (67.3%)
Not documented	5 (3.5%)

**Table 3:** Correlation between the level of protein expression of mesenchymal markers and the clinicopathological criteria.

Clinicopathological Criteria	Snail		Twist		N-Cadherin		Vimentin	
	Positive *N* (%)	Negative *N* (%)	Positive *N* (%)	Negative *N* (%)	Positive *N* (%)	Negative *N* (%)	Positive *N* (%)	Negative *N* (%)
Histological subtype								
IDC ILC UC-MLC	25 (21.7%) 1 (9%) 0 (0%)	90 (78.3%) 11 (91%) 4 (100%)	52 (45.2%) 8 (61.5%) 0 (0%)	63 (54.8%) 5 (38.5%) 4 (100%)	15 (12.8%) 0 (0%) 1 (25%)	102 (87.2%) 13 (100%) 3 (75%)	28 (25.2%) 0 (0%) 2 (50%)	83 (74.8%) 14 (100%) 2 (50%)
		*p* = 0.325		*p* = 0.95		*p* = 0.285		*p* = 0.048
Histological grade								
Grade I Grade II Grade III	3 (12.5%) 7 (14.5%) 16 (38%)	21 (87.5%) 41 (85.5%) 42 (62%)	12 (44.4%) 25 (53.2%) 23 (39.6%)	15 (55.6%) 22 (46.8%) 35 (60.4%)	1 (3.8%) 3 (6.7%) 12 (20%)	25 (96.2%) 45 (93.3%) 48 (80%)	3 (10.4%) 1 (2.2%) 26 (48.2%)	26 (89.6%) 45 (97.8%) 28 (51.8%)
		*p* = 0.149		*p* = 0.380		*p* = 0.033		*p* < 0.0001
Molecular subtype								
Luminal A andB Her2^+^ Triple-negative	12 (15%) 2 (22.2%) 12 (29.3%)	68 (85%) 7 (77.8%) 29 (70.6%)	40 (48.8%) 4 (40%) 16 (40%)	42 (51.2%) 6 (60%) 24 (60%)	3 (3.7%) 2 (20%) 11 (26.8%)	79 (96.3%) 8 (80%) 30 (73.2%)	4 (4.8%) 1 (12.5%) 25 (65.8%)	79 (95.2%) 7 (87.5%) 13 (34.2%)
		*p* = 0.176		*p* = 0.617		*p* = 0.001		*p* < 0.0001
Lymph-node metastasis								
Yes No	9 (21.9%) 16 (18.8%)	32 (78.1%) 69 (81.2%)	21 (51.2%) 36 (41.9%)	20 (48.8%) 50 (58.1%)	2 (4.8%) 14 (17.9%)	40 (95.2%) 73 (82.1%)	7 (18.4%) 22 (24.7%)	31 (81.6%) 67 (75.3%)
		*p* = 0.86		*p* = 0.42		*p* = 0.12		*p* = 0.58

IDC: infiltrating ductal carcinoma; ILC: infiltrating lobular carcinoma; UC-MLC: undifferentiated medullary-like carcinoma; *p* < 0.05 was considered statistically significant.

**Table 4:** Correlation between the level of protein expression of epithelial markers and clinicopathological criteria.

Clinicopathological Criteria	E-Cadherin			GRHL2		EpCAM	
	Positive *N* (%)	Reduced *N* (%)	Negative *N* (%)	Positive *N* (%)	Negative *N* (%)	Positive *N* (%)	Negative *N* (%)
Histological subtype							
IDC ILC UC-MLC	75 (61.5%) 0 (0%) 1 (25%)	44 (36%) 4 (28.6%) 3 (75%)	3 (2.5%) 10 (71.4%) 0 (0%)	109 (90.8%) 14 (100%) 4 (100%)	11 (9.2%) 0 (0%) 0 (0%)	40 (33.3%) 0 (%) 2 (50%)	80 (66.7%) 13 (100%) 2 (50%)
			*p* < 0.001		*p* = 0.408		*p* = 0.032
Histological grade							
Grade I Grade II Grade III	20 (71.4%) 29 (59.2%) 27 (42.8%)	6 (21.4%) 11 (22.4%) 34 (54%)	2 (7.2%) 9 (18.4%) 2 (3.2%)	29 (100%) 48 (100%) 50 (82%)	0 (0%) 0 (0%) 11 (18%)	4 (14.3%) 4 (8.3%) 34 (54%)	24 (85.7%) 44 (91.7%) 27 (46%)
			*p* = 0.001		*p* = 0.001		*p* < 0.001
Molecular subtype							
Luminal A and B Her2^+^ Triple-negative	55 (63.2%) 6 (60%) 15 (34.9%)	21 (24.2%) 4 (40%) 26 (60.5%)	11 (12.6%) 0 (0%) 2 (4.6%)	85 (98.8%) 9 (90%) 33 (78.6%)	1 (1.2%) 1 (10%) 9 (21.4%)	11 (12.9%) 3 (30%) 28 (66.7%)	74 (87.1%) 7 (70%) 14 (33.3%)
			*p* = 0.001		*p* < 0.001		*p* < 0.001
Lymph-node metastasis							
Yes No	20 (47.6%) 53 (57%)	15 (35.7%) 34 (36.6%)	7 (16.7%) 6 (6.4%)	37 (88.1%) 88 (94.6%)	5 (11.9%) 5 (5.4%)	10 (24.4%) 53 (46.5%)	31 (75.6%) 61 (53.5%)
			*p* = 0.16		*p* = 0.32		*p* = 0.02

IDC: infiltrating ductal carcinoma; ILC: infiltrating lobular carcinoma; UC-MLC: undifferentiated medullary-like carcinoma; *p* < 0.05 was considered statistically significant.

**Table 5:** Correlation between the clinicopathological criteria and the degree of the inflammatory infiltrate.

Clinicopathological Criteria	Inflammatory Infiltrate (H&E-Stained WS)		Inflammatory Infiltrate (IHC-Stained TMAs)	
	Mild *N* (%)	Intense *N* (%)	Mild *N* (%)	Intense *N* (%)
Histological subtype				
IDC	100 (79.4%)	26 (20.6%)	84 (75.7%)	27 (24.3%)
ILC	13 (92.8%)	1 (7.2%)	13 (92.8%)	1 (7.2%)
UC-MLC	1 (25%)	3 (75%)	1 (25%)	3 (75%)
	*p* = 0.013		*p* = 0.02	
Histological Grade				
Grade I	31 (100%)	0 (0)	24 (100%)	0 (0)
Grade II	46 (92%)	4 (8%)	44 (93.6%)	3 (6.4%)
Grade III	37 (58.7%)	26 (41.3%)	29 (50.9%)	28 (49.1%)
	*p* < 0.0001		*p* < 0.0001	
Molecular subtype				
Luminal A	64 (98.5%)	1 (1.5%)	58 (100%)	0 (0%)
Luminal B	21 (80.8%)	5 (19.2 5)	16 (76.2%)	5 (23.8)
HER2^+^	4 (40%)	6 (60%)	3 (33.3%)	6 (66.7%)
Triple-negative	25 (58.1%)	18 (41.9%)	20 (52.6%)	18 (47.4%)
	*p* < 0.0001		*p* < 0.0001	
Lymph-node metastasis				
Yes	31 (73.8%)	11 (26.2%)	26 (66.7%)	13 (33.3%)
No	80 (82.5%)	17 (17.5%)	66 (79.5%)	17 (20.5%)
	*p* = 0.34		*p* = 0.19	

IDC: infiltrating ductal carcinoma; ILC: infiltrating lobular carcinoma; UC-MLC: undifferentiated medullary-like carcinoma; WS: whole sections; TMA: tissue microarray; H&E: hematoxylin and eosin; IHC: immunohistochemistry-stained using markers for the most abundant cell populations (CD3, CD20, and MUM1). *p* < 0.05 was considered statistically significant.

**Table 6:** Association between the levels of protein expression of EMT-related markers and the degree of inflammatory infiltrate.

EMT-Related Markers		Infiltrate (H&E-Stained WS)		Infiltrate (IHC-Stained TMAs)	
		Mild	Intense	Mild	Intense
Snail	Negative	82 (78.8%)	22 (21.2%)	75 (78.9%)	20 (21.1%)
	Positive	21 (80.8%)	5 (19.2%)	18 (72%)	7 (28%)
		*p* = 1.000		*p* = 0.769	
Twist	Negative	54 (75%)	18 (25%)	50 (71.4%)	20 (28.6%)
	Positive	50 (83.3%)	10 (16.7%)	44 (83%)	9 (17%)
		*p* = 0.34		*p* = 0.199	
N-cadherin	Negative	94 (79.7%)	24 (20.3%)	85 (76.6%)	26 (23.4%)
	Positive	12 (75%)	4 (25%)	11 (73.3%)	4 (26.6%)
		*p* = 0.92		*p* = 1.000	
Vimentin	Negative	84 (84.8%)	15 (15.2%)	78 (84.8%)	14 (15.2%)
	Positive	20 (66.7%)	10 (33.3%)	16 (57.1%)	12 (42.9%)
		*p* = 0.052		*p* = 0.004	
E-cadherin	Negative	13 (100%)	0 (0%)	12 (92.3%)	1 (7.7%)
	Reduced	33 (64.7%)	18 (35.3%)	29 (63%)	17 (37%)
	Positive	64 (84.2%)	12 (15.8%)	54 (80.5%)	13 (19.5%)
		*p* = 0.005		*p* = 0.03	
GRHL2	Negative	5 (45.4%)	6 (54.6%)	1 (10%)	9 (90%)
	Positive	105 (82%)	23 (18%)	94 (81%)	22 (19%)
		*p* = 0.013		*p* = 0.001	
EpCAM	Negative	80 (84.2%)	15 (15.8%)	76 (83.5%)	15 (16.5%)
	Positive	28 (66.7%)	14 (33.3%)	19 (54.3%)	16 (45.7%)
		*p* = 0.037		*p* < 0.0001	

WS: whole sections; TMA: tissue microarray; H&E: hematoxylin and eosin; IHC: immunohistochemistry-stained using markers for the most abundant cell populations (CD3, CD20, and MUM1). *p* < 0.05 was considered statistically significant.

**Table 7:** Association between the EMT-related markers and the immune cell populations which constitute the inflammatory infiltrate of the TME.

	Snail	Twist	N-Cadherin	Vimentin	E-Cadherin	Ep CAM	GRHL2
CD4/CD8	*p* = 0.62	*p* = 0.24	*p* = 0.004	*p* = 0.25	*p* = 0.34	*p* = 0.52	*p* = 0.33
FOXP3/CD4	*p* = 0.38	*p* = 0.93	*p* = 0.09	*p* = 0.59	*p* = 0.25	*p* = 0.82	*p* = 0.64
CD20/CD3	*p* = 0.15	*p* = 0.53	*p* = 0.72	*p* = 0.05	*p* = 0.35	*p* = 0.03	*p* = 0.29
MUM1	*p* = 0.99	*p* = 0.039	*p* = 0.87	*p* = 0.001	*p* = 0.005	*p* = 0.008	*p* = 0.03

*p* < 0.05 was considered statistically significant.

**Table 8:** Association between the different groups and the degree of inflammatory infiltrate assessed by IHC.

	Inflammatory Infiltrate		*p*-Value
	Mild	Intense	
Group 1: E-cadherin positive + absence of expression of 4 mesenchymal markers	39 (84.7%)	7 (15.3%)	*p* = 0.03
Group 2: E-cadherin positive or reduced + presence of 1 or more mesenchymal markers	32 (65.3%)	17 (34.7%)	
Group 3: E-cadherin negative+ absence of expression of 4 mesenchymal markers	11 (100%)	0 (0%)	
Group 4: E-cadherin reduced + absence of expression of 4 mesenchymal markers	14 (77.8%)	4 (22.2%)	

*p* < 0.05 was considered statistically significant.

**Table 9:** Association between the patterns of expression of E-cadherin and β-catenin.

		β-Catenin			
		Negative	Membranous	Grade1	Grade2
E-cadherin	Negative	9 (100%)	0	0	0
	Reduced	4 (9.7%)	1 (2.4%)	21 (51.2%)	15(36.6%)
	Positive	0	8 (12.3%)	29 (44.6%)	28 (43.1%)
		*p* < 0.0001 (*p* = 0.02) *			

* *p*-Value between the two groups of reduced and positive E-cadherin.

## Data Availability

The datasets used and/or analyzed during the current study are available from the corresponding author on reasonable request.

## Supplementary Materials

The following are available online at https://www.mdpi.com/article/10.3390/cancers13205099/s1: Figure S1. Immunohistochemical labeling of the mesenchymal markers Snail (A), ZEB1 (B), and N-cadherin (C) in breast cancer; Figure S2-I. Immunohistochemical labeling of Twist (A), GRHL2 (B), and EpCAM (C) in breast cancer tissue samples and its evaluation using the Histo-score; Figure S2-II. Immunohistochemical labeling of E-cadherin; Figure S3. Immunohistochemical labeling of ZEB1 in three cases of breast cancer; Figure S4. Immunohistochemical labeling of β–catenin.

## References

[1] Viale G. (2012). The current state of breast cancer classification. Ann. Oncol..

[2] Singh S., Chakrabarti R. (2019). Consequences of EMT-Driven Changes in the Immune Microenvironment of Breast Cancer and Therapeutic Response of Cancer Cells. J. Clin. Med..

[3] Chen H.-T., Liu H., Mao M.-J., Tan Y., Mo X.-Q., Meng X.-J., Cao M.-T., Zhong C.-Y., Liu Y., Shan H. (2019). Crosstalk between autophagy and epithelial-mesenchymal transition and its application in cancer therapy. Mol. Cancer.

[4] Wu Y., Sarkissyan M., Vadgama J.V. (2016). Epithelial-Mesenchymal Transition and Breast Cancer. J. Clin. Med..

[5] Lamouille S., Xu J., Derynck R. (2014). Molecular mechanisms of epithelial–mesenchymal transition. Nat. Rev. Mol. Cell Biol..

[6] Bronsert P., Enderle-Ammour K., Bader M., Timme S., Kuehs M., Csanadi A., Kayser G., Kohler I., Bausch D., Hoeppner J. (2014). Cancer cell invasion and EMT marker expression: A three-dimensional study of the human cancer-host interface. J. Pathol..

[7] Kröger C., Afeyan A., Mraz J., Eaton E.N., Reinhardt F., Khodor Y.L., Thiru P., Bierie B., Ye X., Burge C.B. (2019). Acquisition of a hybrid E/M state is essential for tumorigenicity of basal breast cancer cells. Proc. Natl. Acad. Sci. USA.

[8] Godin L., Balsat C., Van Eycke Y.-R., Allard J., Royer C., Remmelink M., Pastushenko I., D’Haene N., Blanpain C., Salmon I. (2020). A Novel Approach for Quantifying Cancer Cells Showing Hybrid Epithelial/Mesenchymal States in Large Series of Tissue Samples: Towards a New Prognostic Marker. Cancers.

[9] Celià-Terrassa T., Jolly M.K. (2020). Cancer Stem Cells and Epithelial-to-Mesenchymal Transition in Cancer Metastasis. Cold Spring Harb. Perspect. Med..

[10] Gupta P.B., Pastushenko I., Skibinski A., Blanpain C., Kuperwasser C. (2019). Phenotypic Plasticity: Driver of Cancer Initiation, Progression, and Therapy Resistance. Cell Stem Cell.

[11] Nieto M.A., Huang R.Y.-J., Jackson R.A., Thiery J.P. (2016). EMT: 2016. Cell.

[12] Terry S., Savagner P., Ortiz-Cuaran S., Mahjoubi L., Saintigny P., Thiery J.-P., Chouaib S. (2017). New insights into the role of EMT in tumor immune escape. Mol. Oncol..

[13] Fischer K.R., Durrans A., Lee S., Sheng J., Li F., Wong S.T.C., Choi H., El Rayes T., Ryu S., Troeger J. (2015). Epithelial-to-mesenchymal transition is not required for lung metastasis but contributes to chemoresistance. Nature.

[14] Brenot A., Knolhoff B.L., DeNardo D.G., Longmore G.D. (2018). SNAIL1 action in tumor cells influences macrophage polarization and metastasis in breast cancer through altered GM-CSF secretion. Oncogenesis.

[15] Cortés M., Sanchez-Moral L., de Barrios O., Fernández-Aceñero M.J., Martínez-Campanario M., Esteve-Codina A., Darling D.S., Győrffy B., Lawrence T., Dean D.C. (2017). Tumor-associated macrophages (TAMs) depend on ZEB1 for their cancer-promoting roles. EMBO J..

[16] Quail D.F., Joyce J.A. (2013). Microenvironmental regulation of tumor progression and metastasis. Nat. Med..

[17] Dongre A., Rashidian M., Reinhardt F., Bagnato A., Keckesova Z., Ploegh H.L., Weinberg R.A. (2017). Epithelial-to-Mesenchymal Transition Contributes to Immunosuppression in Breast Carcinomas. Cancer Res..

[18] Mrozik K.M., Blaschuk O.W., Cheong C.M., Zannettino A.C.W., Vandyke K. (2018). N-cadherin in cancer metastasis, its emerging role in haematological malignancies and potential as a therapeutic target in cancer. BMC Cancer.

[19] Wang Y., Shi J., Chai K., Ying X., Zhou B.P. (2013). The Role of Snail in EMT and Tumorigenesis. Curr. Cancer Drug Targets.

[20] Qin Q., Xu Y., He T., Qin C., Xu J. (2012). Normal and disease-related biological functions of Twist1 and underlying molecular mechanisms. Cell Res..

[21] Lindner P., Paul S., Eckstein M., Hampel C., Muenzner J., Erlenbach-Wuensch K., Ahmed H.P., Mahadevan V., Brabletz T., Hartmann A. (2020). EMT transcription factor ZEB1 alters the epigenetic landscape of colorectal cancer cells. Cell Death Dis..

[22] Zhang Y., Xu L., Li A., Han X. (2019). The roles of ZEB1 in tumorigenic progression and epigenetic modifications. Biomed. Pharmacother..

[23] Cieply B., Farris J., Denvir J., Ford H.L., Frisch S.M. (2013). Epithelial–Mesenchymal Transition and Tumor Suppression Are Controlled by a Reciprocal Feedback Loop between ZEB1 and Grainyhead-like-2. Cancer Res..

[24] Spizzo G., Fong D., Wurm M., Ensinger C., Obrist P., Hofer C., Mazzoleni G., Gastl G., Went P. (2011). EpCAM expression in primary tumour tissues and metastases: An immunohistochemical analysis. J. Clin. Pathol..

[25] Chen Z., Fang Z., Ma J. (2021). Regulatory mechanisms and clinical significance of vimentin in breast cancer. Biomed. Pharmacother..

[26] Wellenstein M.D., De Visser K.E. (2018). Cancer-Cell-Intrinsic Mechanisms Shaping the Tumor Immune Landscape. Immunity.

[27] Kononen J., Bubendorf L., Kallioniemi O., Bärlund M., Schraml P., Leighton S., Torhorst J., Mihatsch M.J., Sauter G., Kallionimeni O.-P. (1998). Tissue microarrays for high-throughput molecular profiling of tumor specimens. Nat. Med..

[28] Eble J.N., Tavassoli F.A., Devilee P. (2003). World health organization classification of tumours. Pathology and Genetics Tumours of the Breast and Female Genital Organs.

[29] Kondov B., Milenkovikj Z., Kondov G., Petrushevska G., Basheska N., Bogdanovska-Todorovska M., Tolevska N., Ivkovski L. (2018). Presentation of the Molecular Subtypes of Breast Cancer Detected By Immunohistochemistry in Surgically Treated Patients. Open Access Maced. J. Med. Sci..

[30] Goldhirsch A., Winer E.P., Coates A.S., Gelber R.D., Piccart-Gebhart M., Thürlimann B., Senn H.J., Panel Members (2013). Personalizing the treatment of women with early breast cancer: Highlights of the St Gallen International Expert Consensus on the Primary Therapy of Early Breast Cancer 2013. Ann Oncol..

[31] Denkert C., Wienert S., Poterie A., Loibl S., Budczies J., Badve S., Bago-Horvath Z., Bane A., Bedri S., Brock J. (2016). Standardized evaluation of tumor-infiltrating lymphocytes in breast cancer: Results of the ring studies of the international immuno-oncology biomarker working group. Mod. Pathol..

[32] Nederlof I., De Bortoli D., Bareche Y., Nguyen B., De Maaker M., Hooijer G.K.J., Buisseret L., Kok M., Smid M., Van den Eynden G.G. (2019). Comprehensive evaluation of methods to assess overall and cell-specific immune infiltrates in breast cancer. Breast Cancer Res..

[33] Elmoneim H.M.A., Zaghloul N.M. (2011). Expression of e-cadherin, n-cadherin and snail and their correlation with clinicopathologicalvariants: An immunohistochemical study of 132 invasive ductal breast carcinomas in Egypt. Clinics.

[34] Siitonen S.M., Kononen J.T., Helin H.J., Rantala I.S., Holli K.A., Isola J.J. (1996). Reduced E-Cadherin Expression is Associated With Invasiveness and Unfavorable Prognosis in Breast Cancer. Am. J. Clin. Pathol..

[35] Soysal S., Muenst S., Barbie T., Fleming T., Gao F., Spizzo G., Oertli D., Viehl C.T., Obermann E.C., Gillanders W.E. (2013). EpCAM expression varies significantly and is differentially associated with prognosis in the luminal B HER2+, basal-like, and HER2 intrinsic subtypes of breast cancer. Br. J. Cancer.

[36] Hemalatha A., Suresh T.N., Kumar M.H. (2013). Expression of vimentin in breast carcinoma, its correlation with Ki67 and other histopathological parameters. Indian J. Cancer.

[37] Borcherding N., Cole K., Kluz P., Jorgensen M., Kolb R., Bellizzi A., Zhang W. (2018). Re-Evaluating E-Cadherin and β-Catenin: A Pan-Cancer Proteomic Approach with an Emphasis on Breast Cancer. Am. J. Pathol..

[38] Varma K., Chauhan A., Bhargava M., Misra V., Srivastava S. (2020). Association of different patterns of expression of beta-catenin and cyclin D1 with pathogenesis of breast carcinoma. Indian J. Pathol. Microbiol..

[39] Stanton S.E., Disis M.L. (2016). Clinical significance of tumor-infiltrating lymphocytes in breast cancer. J. Immunother. Cancer.

[40] Jézéquel P., Kerdraon O., Hondermarck H., Guérin-Charbonnel C., Lasla H., Gouraud W., Canon J.-L., Gombos A., Dalenc F., Delaloge S. (2019). Identification of three subtypes of triple-negative breast cancer with potential therapeutic implications. Breast Cancer Res..

[41] Barnes T.A., Amir E. (2017). HYPE or HOPE: The prognostic value of infiltrating immune cells in cancer. Br. J. Cancer.

[42] Gruosso T., Gigoux M., Manem V.S.K., Bertos N., Zuo D., Perlitch I., Saleh S.M.I., Zhao H., Souleimanova M., Johnson R.M. (2019). Spatially distinct tumor immune microenvironments stratify triple-negative breast cancers. J. Clin. Investig..

[43] Van Rooijen J.M., Stutvoet T.S., Schröder C.P., de Vries E. (2015). Immunotherapeutic options on the horizon in breast cancer treatment. Pharmacol. Ther..

[44] Jiang X., Ellison S., Alarid E.T., Shapiro D.J. (2007). Interplay between the levels of estrogen and estrogen receptor controls the level of the granzyme inhibitor, proteinase inhibitor 9 and susceptibility to immune surveillance by natural killer cells. Oncogene.

[45] Mostafa A., Codner D., Hirasawa K., Komatsu Y., Young M.N., Steimle V., Drover S. (2014). Activation of ERα Signaling Differentially Modulates IFN-γ Induced HLA-Class II Expression in Breast Cancer Cells. PLoS ONE.

[46] Sahoo S., Nayak S.P., Hari K., Purkait P., Mandal S., Kishore A., Levine H., Jolly M.K. (2021). Immunosuppressive traits of the hybrid epithelial/mesenchymal phenotype. bioRxiv.

[47] Binnewies M., Roberts E.W., Kersten K., Chan V., Fearon D.F., Merad M., Coussens L.M., Gabrilovich D.I., Ostrand-Rosenberg S., Hedrick C.C. (2018). Understanding the tumor immune mi-croenvironment (TIME) for effective therapy. Nat. Med..

[48] Lim B., Woodward W., Wang X., Reuben J.M., Ueno N.T. (2018). Inflammatory breast cancer biology: The tumour microenvironment is key. Nat. Rev. Cancer.

[49] Yeong J., Lim J.C., Lee B., Li H., Chia N., Ong C.C., Lye W.K., Putti T.C., Dent R., Lim E. (2018). High densities of tumor-associated plasma cells predict improved prognosis in triple negative breast cancer. Front. Immunol..

[50] Brown J.R., Wimberly H., Lannin D.R., Nixon C., Rimm D.L., Bossuyt V. (2014). Multiplexed Quantitative Analysis of CD3, CD8, and CD20 Predicts Response to Neoadjuvant Chemotherapy in Breast Cancer. Clin. Cancer Res..

[51] Bar I., Theate I., Haussy S., Beniuga G., Carrasco J., Canon J.-L., Delrée P., Merhi A. (2020). MiR-210 Is Overexpressed in Tumor-infiltrating Plasma Cells in Triple-negative Breast Cancer. J. Histochem. Cytochem..

[52] Wouters M.C.A., Nelson B.H. (2018). Prognostic significance of tumor-infiltrating B cells and plasma cells in human cancer. Clin. Cancer Res..

[53] Shen M., Wang J., Ren X. (2018). New Insights into Tumor-Infiltrating B Lymphocytes in Breast Cancer: Clinical Impacts and Regulatory Mechanisms. Front. Immunol..

[54] MadorskyRowdo F.P., Baron A., Urrutia M., Mordoh J. (2015). Immunotherapy in cancer: A combat between tumors and the immune system; you win some, you lose some. Front. Immunol..

[55] Ding Z.C., Zhou G. (2012). Cytotoxic chemotherapy and CD4+ effector T cells: An emerging alliance for durable antitumor effects. Clin. Dev. Immunol..

[56] Kristensen V.N., Vaske C., Ursini-Siegel J., Van Loo P., Nordgard S.H., Sachidanandam R., Sorlie T., Wärnberg F., Haakensen V.D., Helland Å. (2012). Integrated molecular profiles of invasive breast tumors and ductal carcinoma in situ (DCIS) reveal differential vascular and interleukin signaling. Proc. Natl. Acad. Sci. USA.

[57] Emens L.A. (2012). Breast cancer immunobiology driving immunotherapy: Vaccines and immune checkpoint blockade. Expert Rev. Anticancer. Ther..

[58] DeNardo D.G., Brennan D.J., Rexhepaj E., Ruffell B., Shiao S.L., Madden S.F., Gallagher W.M., Wadhwani N., Keil S.D., Junaid S.A. (2011). Leukocyte Complexity Predicts Breast Cancer Survival and Functionally Regulates Response to Chemotherapy. Cancer Discov..

[59] Chen L., Gibbons D.L., Goswami S., Cortez M.A., Ahn Y.-H., Byers L.A., Zhang X., Yi X., Dwyer D., Lin W. (2014). Metastasis is regulated via microRNA-200/ZEB1 axis control of tumour cell PD-L1 expression and intratumoral immunosuppression. Nat. Commun..

[60] Aiello N., Maddipati R., Norgard R.J., Balli D., Li J., Yuan S., Yamazoe T., Black T., Sahmoud A., Furth E.E. (2018). EMT Subtype Influences Epithelial Plasticity and Mode of Cell Migration. Dev. Cell.

[61] Sahoo S., Mishra A., Kaur H., Hari K., Muralidharan S., Mandal S., Jolly M.K. (2021). A mechanistic model captures the emergence and implications of non-genetic heterogeneity and reversible drug resistance in ER+ breast cancer cells. NAR Cancer.

[62] Kai K., Masuda M., Aishima S. (2017). Inverse correlation between CD8+ inflammatory cells and E-cadherin expression in gallbladder cancer: Tissue microarray and imaging analysis. World J. Clin. Cases.

[63] Chang H.-Y., Tseng Y.-K., Chen Y.-C., Shu C.-W., Lin M.-I., Liou H.-H., Fu T.-Y., Lin Y.-C., Ger L.-P., Yeh M.-H. (2018). High snail expression predicts a poor prognosis in breast invasive ductal carcinoma patients with HER2/EGFR-positive subtypes. Surg. Oncol..

[64] Blanco M.J., Moreno-Bueno G., Sarrio D., Locascio A., Cano A., Palacios J., Nieto M.Á. (2002). Correlation of Snail expression with histological grade and lymph node status in breast carcinomas. Oncogene.

[65] He T., Tan R., Wang L., Song J., Li J. (2017). Expression and significance of Twist, estrogen receptor, and E-cadherin in human breast cancer cells and tissues. J. Cancer Res. Ther..

[66] Berx G., Van Roy F. (2001). The E-cadherin/catenin complex: An important gatekeeper in breast cancer tumorigenesis and malignant progression. Breast Cancer Res..

[67] Acs G., Lawton T.J., Rebbeck T.R., Livolsi V.A., Zhang P.J. (2001). Differential Expression of E-Cadherin in Lobular and Ductal Neoplasms of the Breast and Its Biologic and Diagnostic Implications. Am. J. Clin. Pathol..

[68] Yoshida R., Kimura N., Harada Y., Ohuchi N. (2001). The loss of E-cadherin, alpha- and beta-catenin expression is associated with metastasis and poor prognosis in invasive breast cancer. Int. J. Oncol..

[69] Xiang X., Deng Z., Zhuang X., Ju S., Mu J., Jiang H., Zhang L., Yan J., Miller D., Zhang H.-G. (2012). Grhl2 Determines the Epithelial Phenotype of Breast Cancers and Promotes Tumor Progression. PLoS ONE.

[70] Sorlie T., Perou C.M., Tibshirani R., Aas T., Geisler S., Johnsen H., Hastie T., Eisen M.B., van de Rijn M., Jeffrey S.S. (2001). Gene expression patterns of breast carcinomas distinguish tumor subclasses with clinical implications. Proc. Natl. Acad. Sci. USA.

[71] Münz M., Kieu C., Mack B., Schmitt B., Zeidler R., Gires O. (2004). The carcinoma-associated antigen EpCAM upregulates c-myc and induces cell proliferation. Oncogene.

[72] Winter M.J., Nagelkerken B., Mertens A.E., Rees-Bakker H.A., Bruijn I.H.B.-D., Litvinov S.V. (2003). Expression of Ep-CAM shifts the state of cadherin-mediated adhesions from strong to weak. Exp. Cell Res..

[73] Huelsken J., Behrens J. (2002). The Wntsignalling pathway. J. Cell Sci..

[74] Osta W.A., Chen Y., Mikhitarian K., Mitas M., Salem M., Hannun Y.A., Cole D.J., Gillanders W.E. (2004). EpCAM Is Overexpressed in Breast Cancer and Is a Potential Target for Breast Cancer Gene Therapy. Cancer Res..

[75] Balzar M., Prins F.A., Bakker H.A., Fleuren G.J., Warnaar S.O., Litvinov S.V. (1999). The Structural Analysis of Adhesions Mediated by Ep-CAM. Exp. Cell Res..

[76] Sankpal N.V., Fleming T.P., Sharma P.K., Wiedner H., Gillanders W.E. (2017). A double-negative feedback loop between EpCAM and ERK contributes to the regulation of epithelial–mesenchymal transition in cancer. Oncogene.

[77] Driemel C., Kremling H., Schumacher S., Will D., Wolters J., Lindenlauf N., Mack B., Baldus S.A., Hoya V., Pietsch J.M. (2013). Context-dependent adaption of EpCAM expression in early systemic esophageal cancer. Oncogene.

[78] Markiewicz A., Wełnicka-Jaśkiewicz M., Seroczyńska B., Skokowski J., Majewska H., Szade J., Żaczek A.J. (2014). Epithelial-mesenchymal transition markers in lymph node metastases and primary breast tumors-relation to dissemination and proliferation. Am. J. Transl. Res..

[79] Savci-Heijink C.D., Halfwerk H., Hooijer G.K.J., Koster J., Horlings H.M., Meijer S.L., Van De Vijver M.J. (2019). Epithelial-to-mesenchymal transition status of primary breast carcinomas and its correlation with metastatic behavior. Breast Cancer Res. Treat..

