# Supplementary material for: Impact of Epithelial–Mesenchymal Transition on the Immune Landscape in Breast Cancer
Source: Cancers (Basel). 2021 Oct 12;13(20):5099. doi: 10.3390/cancers13205099 (PMC8533811; doi:10.3390/cancers13205099)
Supplement: Supplementary file 1 [file cancers-13-05099-s001.zip › Supplementary Figures - Cancers.pptx]

## Slide 1
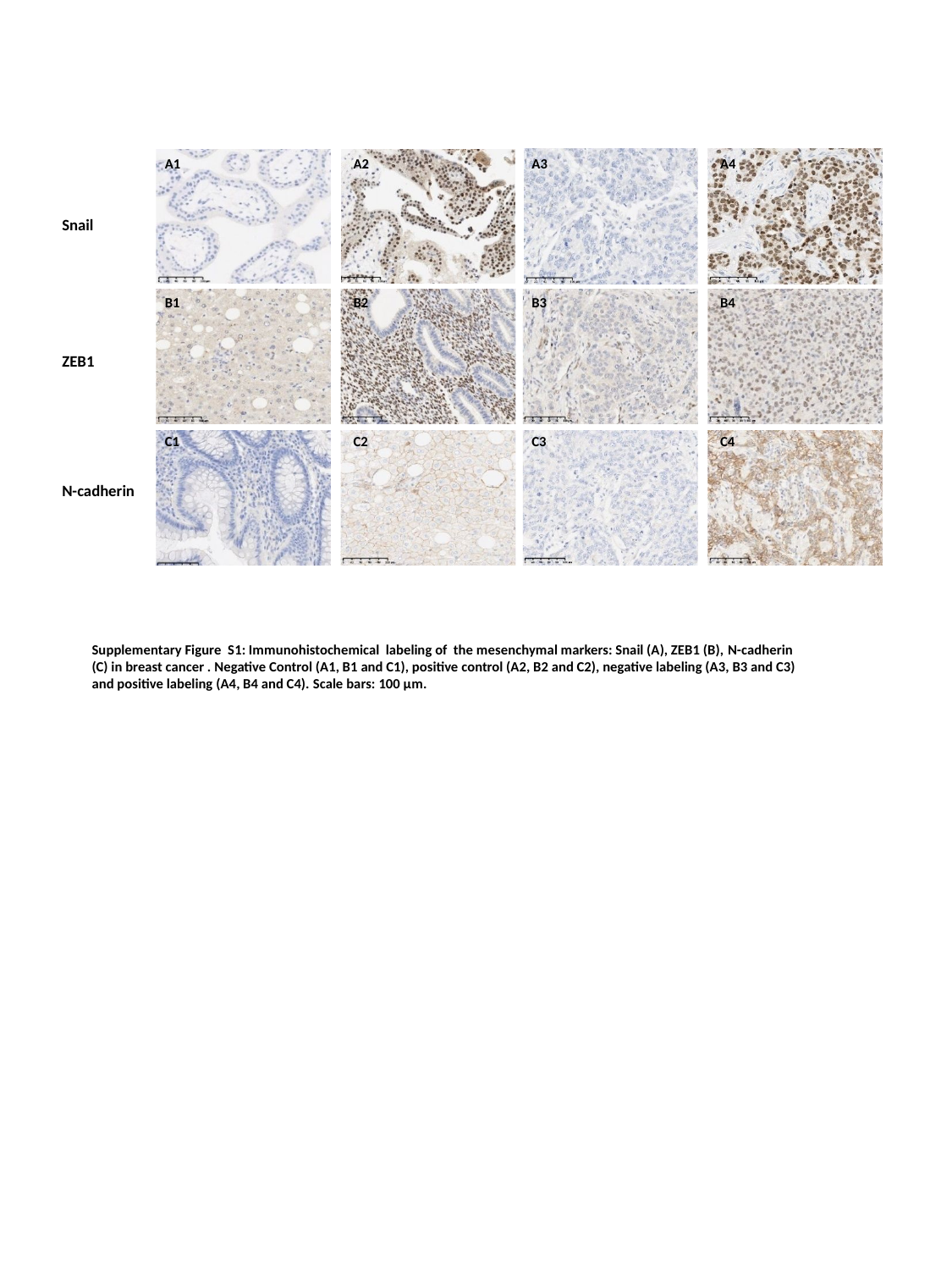

A1
A2
A3
A4
B1
B2
B3
B4
C1
C3
C2
C4
Snail
ZEB1
N-cadherin
Supplementary Figure S1: Immunohistochemical labeling of the mesenchymal markers: Snail (A), ZEB1 (B), N-cadherin (C) in breast cancer . Negative Control (A1, B1 and C1), positive control (A2, B2 and C2), negative labeling (A3, B3 and C3) and positive labeling (A4, B4 and C4). Scale bars: 100 μm.

## Slide 2
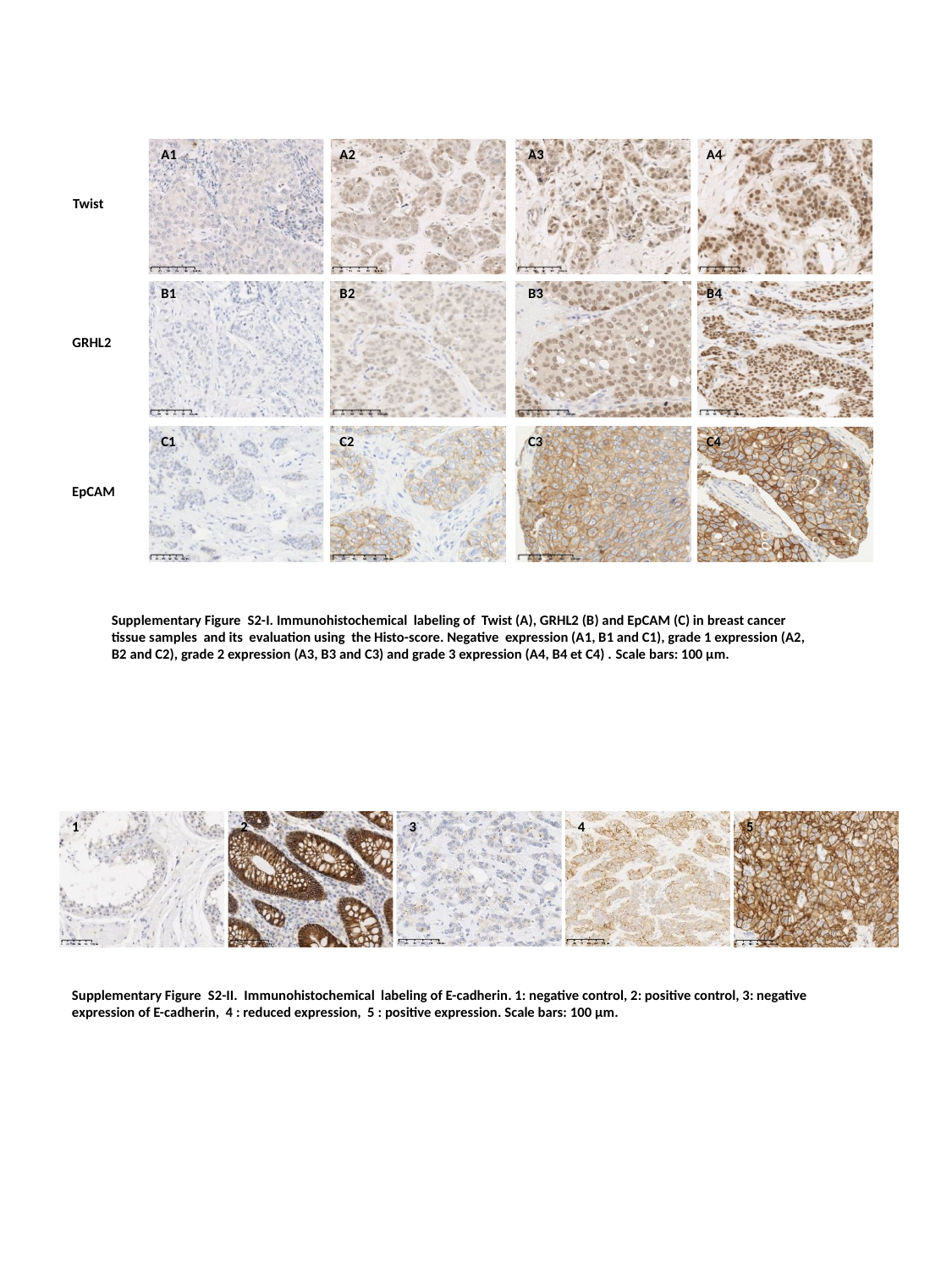

A1
A2
A3
A4
B1
B2
B3
B4
C1
C2
C3
C4
Twist
GRHL2
EpCAM
Supplementary Figure S2-I. Immunohistochemical labeling of Twist (A), GRHL2 (B) and EpCAM (C) in breast cancer tissue samples and its evaluation using the Histo-score. Negative expression (A1, B1 and C1), grade 1 expression (A2, B2 and C2), grade 2 expression (A3, B3 and C3) and grade 3 expression (A4, B4 et C4) . Scale bars: 100 μm.
1
2
3
4
5
Supplementary Figure S2-II. Immunohistochemical labeling of E-cadherin. 1: negative control, 2: positive control, 3: negative expression of E-cadherin, 4 : reduced expression, 5 : positive expression. Scale bars: 100 μm.

## Slide 3
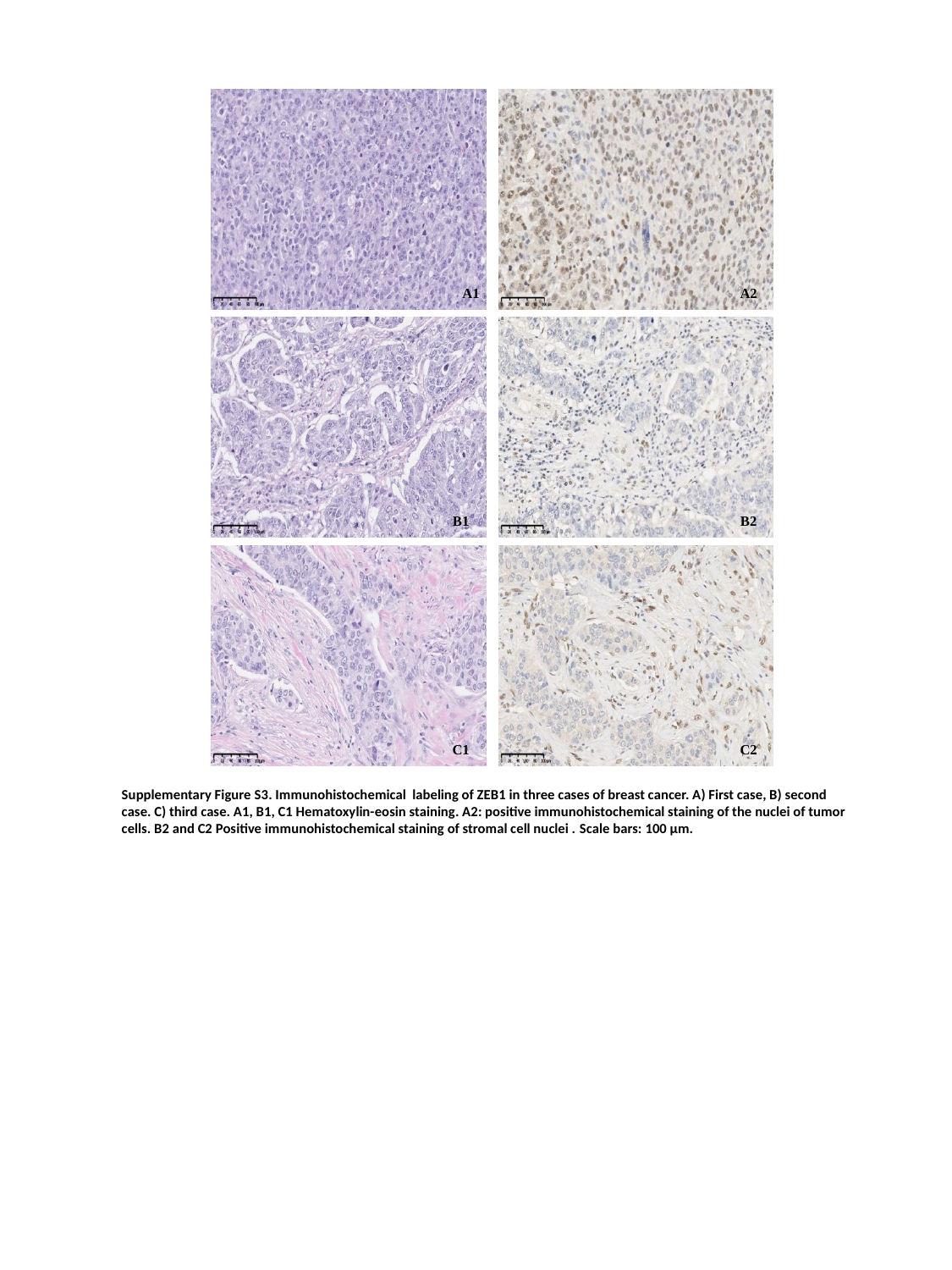

A1
A2
B2
B1
C1
C2
A1
Supplementary Figure S3. Immunohistochemical labeling of ZEB1 in three cases of breast cancer. A) First case, B) second case. C) third case. A1, B1, C1 Hematoxylin-eosin staining. A2: positive immunohistochemical staining of the nuclei of tumor cells. B2 and C2 Positive immunohistochemical staining of stromal cell nuclei . Scale bars: 100 μm.

## Slide 4
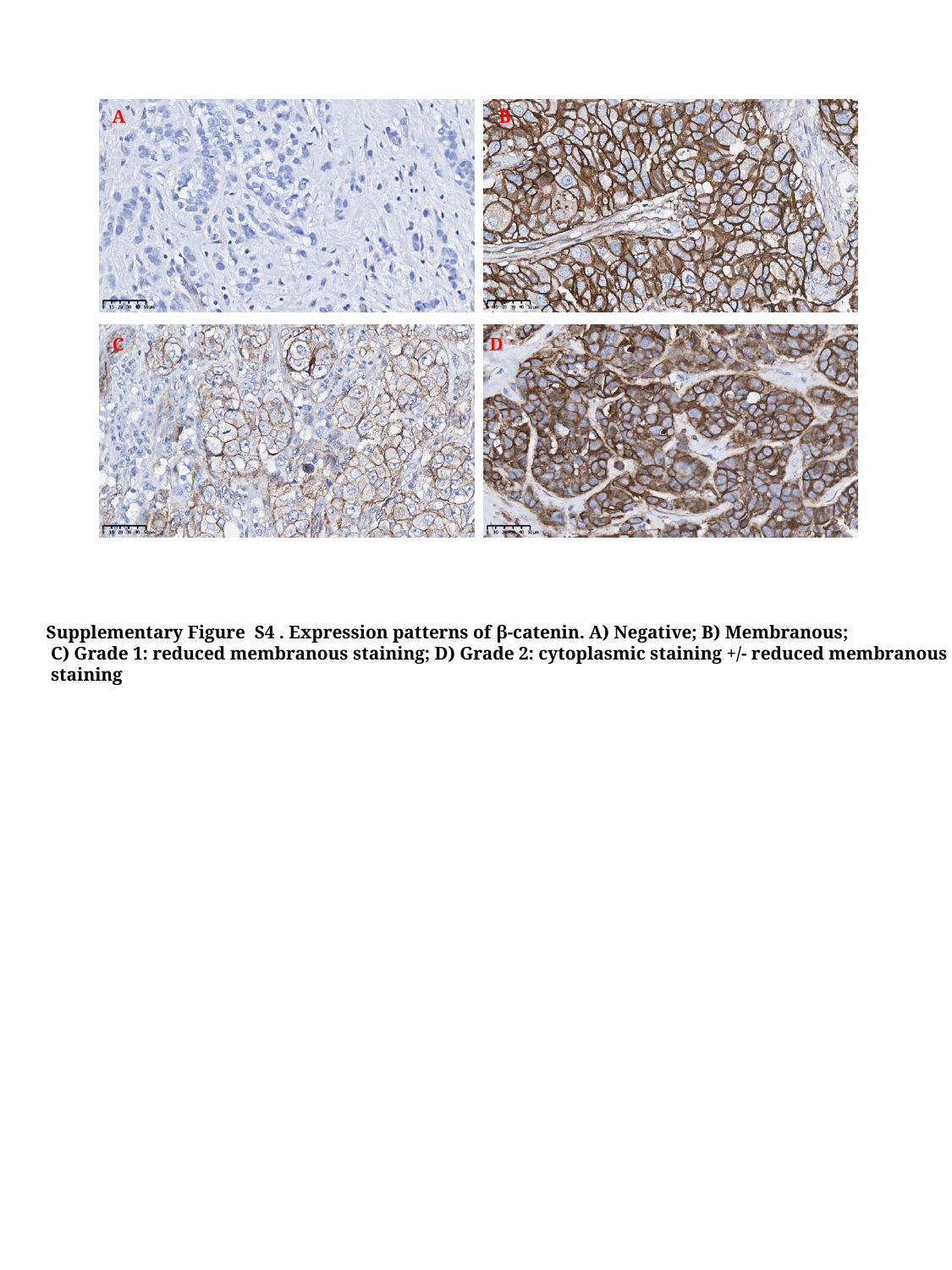

A
B
C
D
Supplementary Figure S4 . Expression patterns of β-catenin. A) Negative; B) Membranous;
 C) Grade 1: reduced membranous staining; D) Grade 2: cytoplasmic staining +/- reduced membranous
 staining
